# The Association Between DNA Methylation and Three-Dimensional Genome During Whole Genome Doubling in *Arabidopsis thaliana*

**DOI:** 10.3390/plants14192959

**Published:** 2025-09-24

**Authors:** Ranze Zhao, Zhongqiu Ni, Dingyu Zhang, Yuda Fang

**Affiliations:** 1 Joint Center for Single Cell Biology, School of Agriculture and Biology, Shanghai Jiao Tong University, Shanghai 200240, China; sn1713283495@163.com (R.Z.); zhongqiu_neau@126.com (Z.N.); 2 Shanghai Key Laboratory of Protected Horticultural Technology, Horticultural Research Institute, Shanghai Academy of Agricultural Sciences, Shanghai 201403, China

**Keywords:** *Arabidopsis thaliana*, Hi-C, WGD, DNA methylation, WGBS

## Abstract

Whole genome doubling (WGD) triggers profound genomic and epigenetic reorganization, yet the functional dynamics of DNA methylation during this process remain incompletely resolved. Here, we integrate whole genome bisulfite sequencing (WGBS) and three-dimensional chromatin interaction data to display methylation landscapes in autotetraploid *Arabidopsis thaliana*. Our analysis reveals evolutionarily conserved spatial patterning of DNA methylation after WGD, with centromeric enrichment and telomeric depletion. Chromosome-level profiling identifies Chromosome 2 as the most highly methylated across CG, CHG, and CHH contexts, while Chromosome 1 shows the lowest methylation. Subcontext methylation analysis uncovers increases in methylation levels in autotetraploid *Arabidopsis thaliana*, most pronounced in the CHH context, yet global distribution patterns remain stable. Comparative methylation profiling around genes and transposable elements (TEs) reveals elevated CHH methylation in autotetraploid gene bodies and flanking regions, whereas TE bodies exhibit minimal changes despite minor flanking hypermethylation. Strikingly, 8% of chromatin compartments were restructured, and B-B interactions weakened in autotetraploid, while DNA methylation remained stable across shifting A/B compartments. Our findings suggest that DNA methylation serves as a resilient epigenetic modification during WGD, even if 3D chromatin architecture undergoes reorganization upon WGD in some degree.

## 1. Introduction

WGD, a pivotal driver of plant evolution and speciation, induces profound genomic and epigenetic reorganization [1,2,3]. The emergence of polyploidy is an important time point in the genomic evolution of animals and flowering plants. As a critical research focus in evolutionary biology, WGD and polyploidy have garnered significant attention [4]. In some horticultural plants, polyploidy is frequently associated with advantageous traits such as enlarged fruits, enhanced stress tolerance, and increased leaf size [5,6]. For instance, in citrus plants, polyploidization improves salt stress resistance through ethylene signaling transduction [7]. Similarly, in Brassica plants, cold tolerance genes exhibit preferential retention, highlighting their adaptive significance during polyploid evolution [8]. Emerging evidence underscores the central role of polyploidy in driving evolutionary processes in plants [2,9], making the elucidation of its underlying mechanisms critically important.

Epigenetics, as modifications that do not alter DNA sequences, can contribute to the transcriptional and post-transcriptional regulation of stress response genes involved in abiotic stress responses [10,11,12]. In recent years, the methods for detecting epigenetic signals have become increasingly diversified. For example, CUT&Tag has emerged as an alternative to ChIP-seq, and techniques like ATAC-seq and WGBS have also been developed [13,14]. DNA methylation, now an important aspect of epigenetics, is often thought to be linked to senescence in animals [15], and more and more researchers are beginning to focus on changes in DNA methylation profiles in response to changes in plant growth and development or environmental adaptation [16,17]. DNA methylation is a highly conserved epigenetic modification that plays critical roles in regulating gene expression, silencing transposons, and directing chromosome interactions in plants [18]. Cytosine methylation occurs in three distinct sequence contexts: CG, CHG, and CHH (where H represents A, T, or C). The dynamic regulation of DNA methylation in plants involves three distinct mechanistic layers. De novo methylation is primarily established through the RNA-directed DNA methylation (RdDM) pathway, with DOMAINS REARRANGED METHYLASE 2 (DRM2) catalyzing site-specific methylation guided by small RNAs [19]. Methylation maintenance, however, exhibits context-dependent specialization: CG sites are preserved by METHYLTRANSFERASE 1 (MET1), whereas CHROMOMETHYLASE 2 (CMT2) and CHROMOMETHYLASE 3 (CMT3) maintain CHG and CHH methylation patterns, respectively [20,21,22]. Counteracting these processes, active DNA demethylation is executed by 5-methylcytosine DNA glycosylases such as REPRESSOR OF SILENCING 1 (ROS1), DEMETER (DME), and DEMETER-LIKE PROTEINs (DML2-3), which dynamically erase epigenetic marks to enable developmental plasticity [11,23,24].

The genome within the nucleus often exhibits specific spatial characteristics rather than a linear arrangement. Chromatin topology can influence the interaction of regulatory elements to affect epigenetic regulation. The three-dimensional genome has emerged as a critical frontier in epigenetics, with growing evidence indicating that dynamic 3D chromatin architecture regulates diverse plant developmental stages and abiotic stress responses [25,26,27]. The core issues of three-dimensional genomics include determining the mechanisms by which chromatin folds and compresses within the cell nucleus, as well as the mechanisms by which three-dimensional chromatin structure affects gene expression and cell fate. In 2009, the first generation of high-throughput chromosome conformation capture technology emerged [27], and since then, there has been explosive development both at the technical level and at the data analysis level. Among them, the most important factors influencing biological processes remain no more than the analyses of three different resolutions of A/B compartments, TADs, and loops [28]. A Compartment is mainly located in the central region of the cell nucleus, featuring an open chromatin structure, active histone modification and high GC content. On the contrary, B Compartment is close to the nuclear membrane of the cell and has inhibitory histone modification and high AT content [29]. Tads are regions in the genome where intra-regional contacts are more abundant than inter-regional contacts. The position of the boundary of the TAD is often considered by researchers as an important three-dimensional structural feature, for example, in soybean where different tissues regulate the formation and maintenance of the TAD boundary in response to light by different mechanisms [30]. Loop structures play an important role in regulating promoter-enhancer interactions characterized as the highest dynamic level at high resolution [31]. Our prior work demonstrated that WGD modulates gene expression by altering chromatin loops and compartmental interactions [9]. Hi-C data analysis across varying resolutions reveals compartmental changes influencing heterosis in *Brassica napus* [32,33], TADs contributing to evolutionary conservation and genome stability [34], and chromatin loops linking specific genes, as exemplified by PRC1-mediated H2AK121ub facilitating local and long-range interactions in *Arabidopsis thaliana* [35]. However, the interplay between DNA methylation and 3D genome reorganization during WGD remains unexplored. Previous studies in polyploid crops, such as pakchoi [36], have hinted at conserved methylation patterns post-WGD, yet systematic analyses in model systems like *Arabidopsis thaliana* are limited. Given its well-annotated genome and historical WGD events, *Arabidopsis thaliana* provides a model framework for studying methylation dynamics following genome doubling. Here, we address these gaps by integrating WGBS and 3D chromatin interaction data to dissect the epigenetic landscape of autotetraploid Arabidopsis. Our findings highlight DNA methylation’s stability during duplication.

## 2. Results

### 2.1. Genome-Wide DNA Methylation Landscapes in Arabidopsis thaliana After Whole Genome Doubling

**Figure 1:**
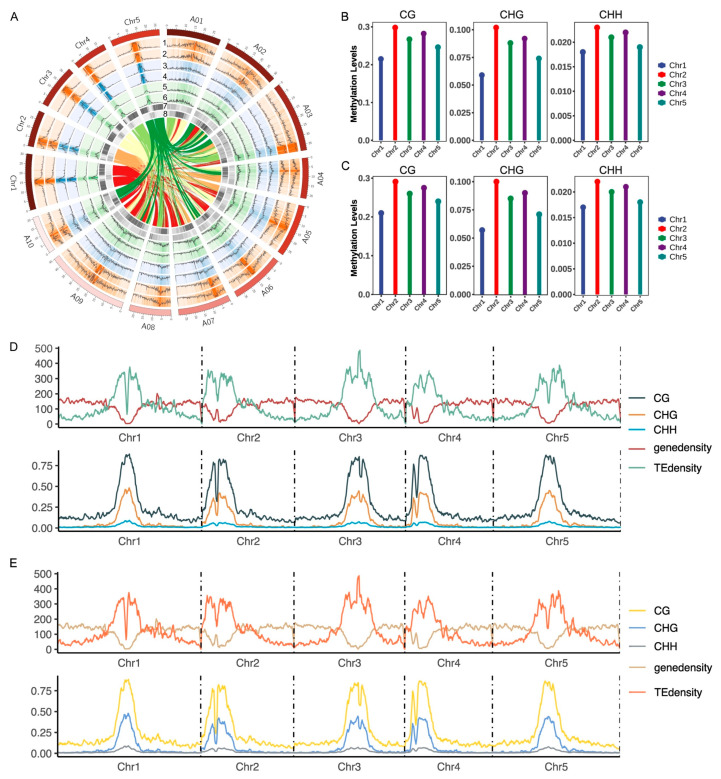
DNA methylation landscape of the Ara2X (diploid Arabidopsis) and Ara4X (autotetraploid Arabidopsis). (**A**) Circos plot showing the gene density and DNA methylation level of CG, CHG, and CHH contexts in the Ara and Bra. (1), (3), (5): DNA methylation levels at CHH, CHG, and CG contexts in the Ara4X and Bra4X; (2), (4), (6): DNA methylation levels in the CHH, CHG, and CG contexts in the Ara2X and Bra2X.; (7): gene density; (8): TE density. The connecting lines in the middle of the Circos plot represent homologous genes between pakchoi and Arabidopsis. (**B**) The distribution of DNA methylation on different chromosomes for three sequence contexts (CG, CHG, CHH) of Ara2X. (**C**) The distribution of DNA methylation on different chromosomes for three sequence contexts (CG, CHG, CHH) of Ara4X. (**D**) DNA methylation in three sequence contexts (CG, CHG, CHH) of Ara2X and the densities of genes and TEs across chromosomes. (**E**) DNA methylation in three sequence contexts (CG, CHG, CHH) of Ara4X and the densities of genes and TEs across chromosomes.

**Figure 2:**
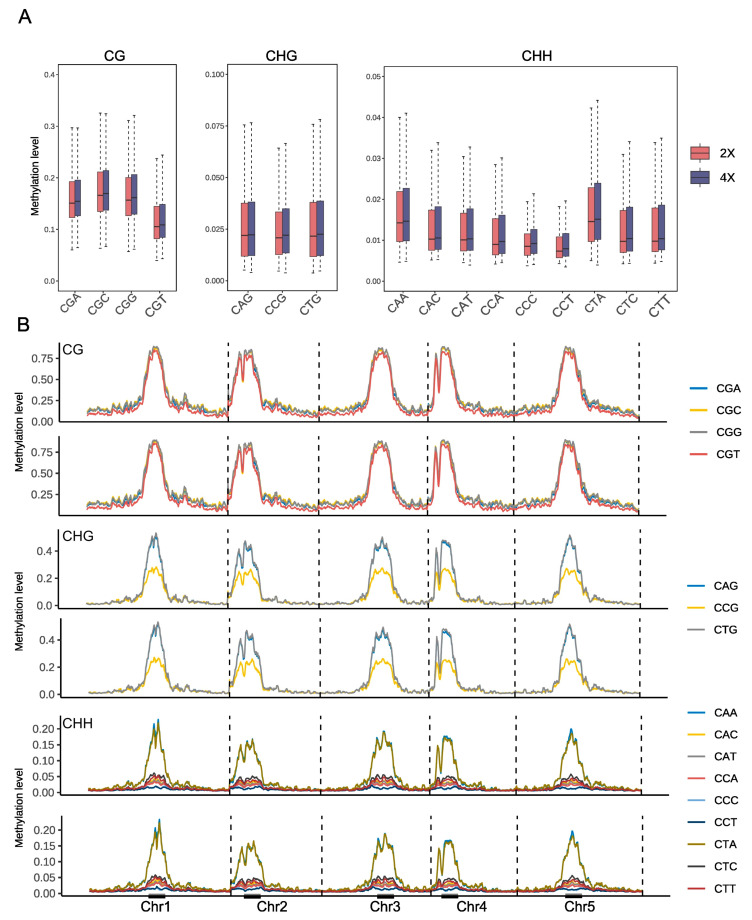
DNA methylation distributions across different subcontexts in the CG, CHG and CHH contexts. (**A**) Methylation levels of each subcontext under CG, CHG and CHH contexts across chromosomes per 100 kb bin in Ara2X and Ara4X. (**B**) Methylation levels of subcontexts across chromosomes per 100 kb bin (upper panel is Ara2X, lower panel is Ara4X).

WGBS was conducted on diploid Arabidopsis and autotetraploid Arabidopsis. We designed two biological replicates per sample, employing paired-end sequencing to achieve a mean depth of 30× genomic coverage. This approach generated 20 M high-quality reads (Q30 > 90%) per sample, of which >70% demonstrated alignment to the TAIR10 reference (Table S1). Consistent with prior findings in polyploid pakchoi, our analysis revealed evolutionarily conserved methylation patterning in diploid and autotetraploid Arabidopsis, characterized by a predominant enrichment in centromeric regions and significant decrease in telomeric regions (Figure 1A,D,E and Figure S1). This conserved spatial organization persists in post-whole genome doubling (WGD), demonstrating that the fundamental epigenetic architecture remains stable despite genomic shock induced by polyploidization. Next, we focused on analyzing methylation levels across individual chromosomes before and after genome doubling. Our examination revealed that across all three methylation contexts (CG, CHG, CHH), Chromosome 2 consistently exhibited the highest average methylation levels, while Chromosome 1 showed the lowest (Figure 1B,C). Further subdivision of each methylation context into specific sequence subcontexts revealed that compared to diploid Arabidopsis, autotetraploid Arabidopsis exhibited a slight increase in methylation levels across all subcontexts, with the most pronounced changes observed in CHH contexts relative to CG and CHG (Figure 2A). While CHG subcontexts (CAG, CCG, CTG) showed minimal variation, CG subcontexts displayed significant divergence, where CGA, CGC and CGG methylation levels markedly exceeded those of CGT. In CHH contexts, CAA and CTA subcontexts exhibited significantly higher methylation levels than other methylation subcontexts (Figure 2B). WGD events represent a massive genomic shock that may disrupt the silencing control of TEs. TE regions are crucial for maintaining genomic stability, and their epigenetic state is highly sensitive to any disruption to prevent potential transposon outbreaks and genomic instability. Notably, the overall distribution patterns across subcontexts remained highly consistent between ploidy states, further supporting the role of DNA methylation in maintaining stability during whole-genome duplication.

### 2.2. DNA Methylation Changes upon Genome Duplication Around Gene and TE

**Figure 3:**
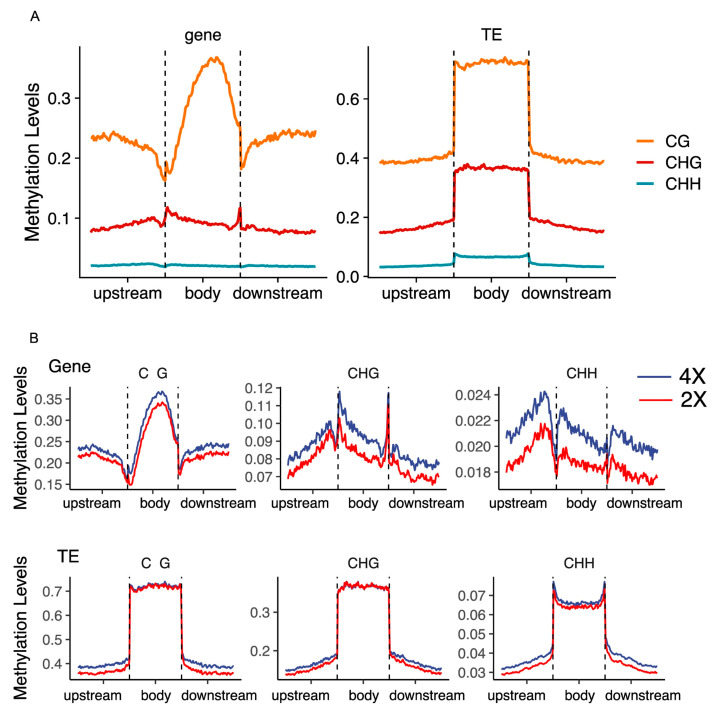
Comparative analysis between Ara2X and Ara4X. (**A**) DNA methylations levels from gene body and TE regions at CG, CHG, and CHH sequence contexts in Ara2X. (**B**) Comparative analysis of DNA methylations levels between Ara2X and Ara4X.

To further investigate the genomic methylation landscape, we compared the average methylation levels around genes and transposable elements (TEs) between diploid and autotetraploid Arabidopsis. The results demonstrated a sharp decrease in DNA methylation levels near transcription start sites (TSSs) and transcription termination sites (TTSs), with elevated CG methylation in gene bodies but relatively lower CHG and CHH methylation (Figure 3A). While the overall methylation distribution patterns around TEs were similar to those near genes, TE bodies exhibited significantly higher methylation levels compared to their flanking 2 kb regions (Figure 3A). Comparative analysis revealed that autotetraploid Arabidopsis showed increased average methylation levels in gene bodies and adjacent regions across all contexts, with the most pronounced differences observed in CHH methylation (Figure 3B). GO analysis of the DMGs suggests that CHH methylation changes may be associated with chromatin organization, small molecule binding, and organelle organization (Figure S2). In contrast, TE bodies displayed minimal changes in methylation levels post-doubling across all contexts, though minor increases were detected in their flanking regions (Figure 3B). These findings suggest that genome doubling in Arabidopsis preferentially enhances methylation near genes while exerting limited effects on TEs, implying a potential role of DNA methylation in suppressing TE transposition activity.

### 2.3. Characterization of Differential Methylation Sites (DMCs) and Differential Methylation Regions (DMRs) at Diploid and Autotetraploid Arabidopsis

**Figure 4:**
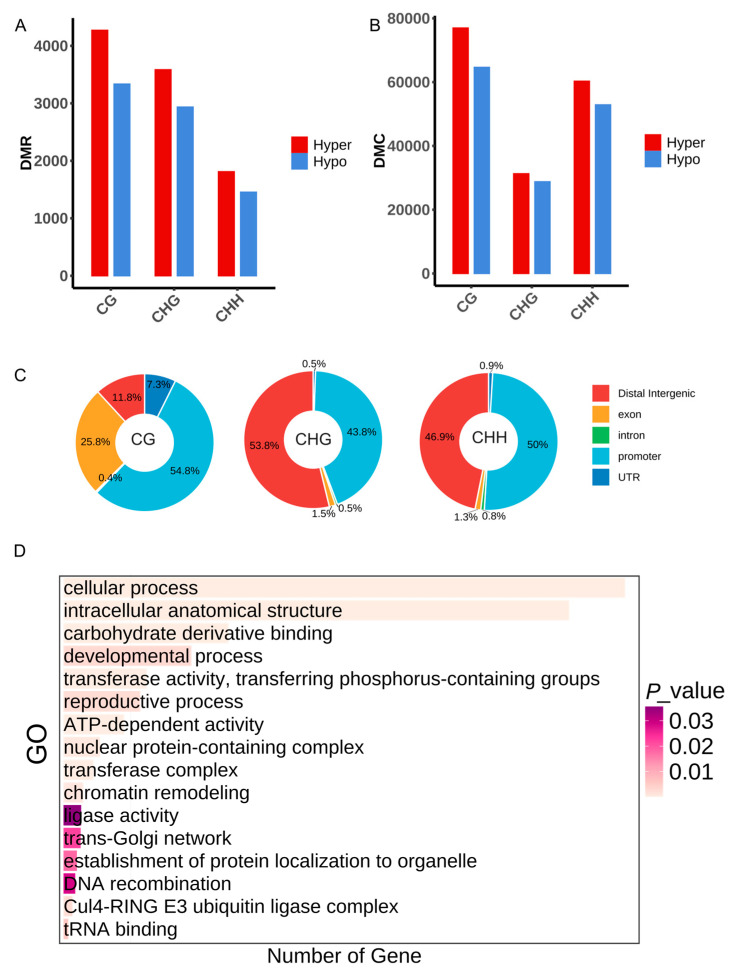
Different methylation analysis between Ara2X and Ara4X. (**A**) Numbers of DMRs between Ara2X and Ara4X. (**B**) Numbers of DMCs between Ara2X and Ara4X. (**C**) Genome-wide Distribution of DMRs. (**D**) Gene Ontology (GO) analysis of DMGs.

Building upon these observations, we performed differential methylation analysis between diploid and autotetraploid Arabidopsis, identifying numerous differentially methylated regions (DMRs) and cytosines (DMCs). In total, we identified 17,388 DMRs, including 4270 hyper-methylated CG-DMRs, 3335 hypo-methylated CG-DMRs, 3584 hyper-methylated CHG-DMRs, 2935 hypo-methylated CHG-DMRs, 1810 hyper-methylated CHH-DMRs, and 1454 hypo-methylated CHH-DMRs, respectively (Figure 4A). This reveals substantial localized methylation divergence despite limited global variation, with hyper-DMRs/DMCs significantly exceeding hypo-DMRs/DMCs, consistent with the higher global methylation levels in autotetraploid Arabidopsis. Genomic distribution analysis demonstrated distinct context-specific patterns: CG-type DMRs predominantly localized to exons, whereas DMRs in CHG and CHH were enriched in promoters and intergenic regions (Figure 4C). Gene ontology (GO) enrichment analysis of these DMRs highlighted functional associations with chromatin remodeling, reproductive processes, and core cellular pathways, suggesting coordinated epigenetic regulation during genome doubling (Figure 4D).

### 2.4. Methylation Levels Remain Stable Across Varying Compartments and TADs

**Figure 5:**
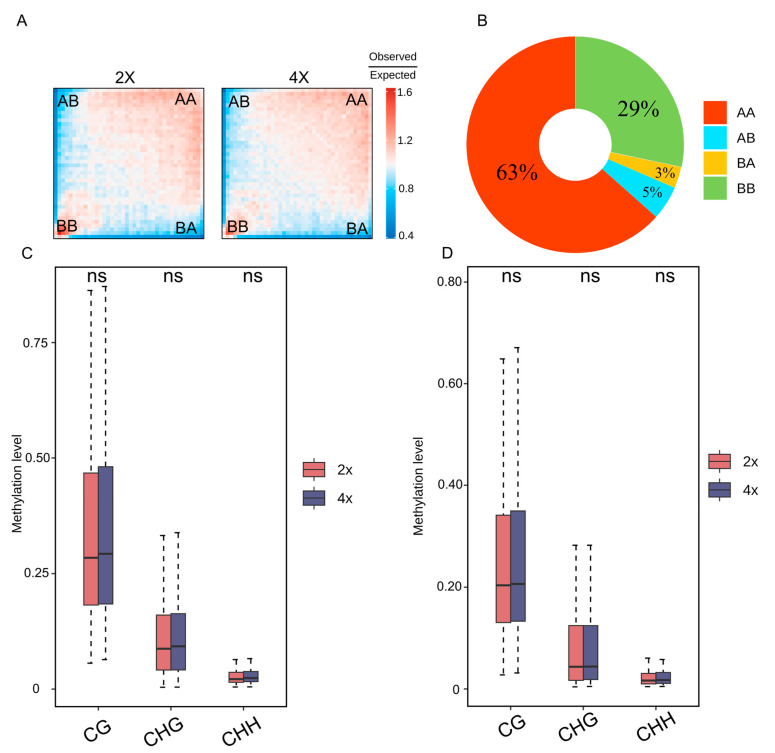
Interplay between DNA methylation and chromatin compartment shifts during genome duplication. (**A**) Saddle plot of compartment strength in Ara2X and Ara4x. (**B**) The distribution of changed compartment during genome duplication. (**C**) DNA methylation level in A-to-B compartment. (**D**) DNA methylation level in B-to-A compartment. Ns represents no significant difference (*t* test).

We first analyzed genome-wide interaction signals and observed no significant chromosomal-level structural alterations during WGD (Figure S1). Based on preliminary laboratory studies indicating that approximately 8% of compartments undergo changes before and after genome doubling (Figure 5B), we further investigated previously unexplored relationships between compartments. The results revealed that, compared to diploid Arabidopsis thaliana, the doubled genome exhibited distinct interaction patterns between compartments, with the most notable being a reduction in B-B compartment interaction strength (Figure 5A). To explore whether DNA methylation on these dynamically altered compartments would change alongside three-dimensional genome reorganization, we analyzed methylation levels in shift compartments (Figure 5C,D). The findings demonstrated no significant changes in DNA methylation levels, either in A-to-B or B-to-A compartments, suggesting that methylation remains stable even within dynamically restructured compartments. Our study indicates that genome doubling induces alterations in chromatin three-dimensional architecture, but DNA methylation does not significantly vary in response to compartmental changes. Although the existence of TADs in compact plant genomes was historically debated, advancements in Hi-C methodologies and analytical algorithms have identified TAD-like domains in Arabidopsis. The regulation of plant gene transcription and epigenetics by TADs has been repeatedly demonstrated [37,38]. We initially identified 925 and 1052 topologically associating domains (TADs) in diploid and autotetraploid Arabidopsis thaliana, respectively, and discovered distributional differences between them (Figure S4). Comparative analysis of TAD regions between diploid and autotetraploid Arabidopsis revealed patterns analogous to compartment reorganization, while the majority of TADs remained conserved, structural variations including Split, Complex, Merge, and Shifted types were identified (Figure S5). Then, we specifically examined DNA methylation dynamics at TAD-like domains versus boundaries during genome doubling. As hypothesized, methylation levels at TAD-like domains significantly exceeded those within boundaries, a pattern conserved post-doubling. Integrated with compartment reorganization analyses, our results demonstrate that genome doubling drives 3D chromatin architectural changes, while DNA methylation exhibits resilience, likely contributing to 3D genomic stabilization.

## 3. Discussion

This study systematically investigates the dynamic changes in DNA methylation and its interplay with 3D genome architecture following WGD in *Arabidopsis thaliana*.

Polyploidy research provides critical insights into species origination, evolutionary relationships, and mechanistic dissection of genomic adaptation [39,40,41]. The effects of DNA methylation dynamics during polyploidization and their functional contributions to plant adaptation remain poorly characterized, despite limited investigations in allopolyploid systems such as wheat and cotton [42,43]. As a model plant with ancestral WGD events since angiosperm evolution, we employed colchicine-induced autotetraploid Arabidopsis to analyze WGD effects [9]. Despite WGD-induced genomic shock, the global spatial methylation patterns—centromeric enrichment and telomeric hypomethylation—remained highly conserved between diploid and tetraploid Arabidopsis (Figure 1C,D), demonstrating robust resilience of core epigenetic architecture during genome duplication. This stability may buffer polyploidy-driven genomic instability by suppressing transposon activity or preserving chromatin compartment integrity. Extensive studies have established CHH methylation as the predominant form in plants [11,44,45,46]. For example, reduced CHH methylation levels affected SOC1 gene expression in moso bamboo flowering [47]. In *Arabidopsis thaliana*, CHH methylation progressively accumulates during embryogenesis [48] and regulates flowering time [47], fruit morphogenesis [46]. Notably, our results consistently demonstrate that CHH methylation exhibits pronounced dynamics during genome doubling compared to CG and CHG contexts. Localized methylation variations, notably CHH context elevation (Figure 2A,B), suggest sequence-specific sensitivity to dosage effects. Post-WGD enhancement of methylation in gene bodies and flanking regions, particularly in CHH contexts (Figure 3B), may underlie polyploid adaptive regulation. CHH hypermethylation likely engages the RNA-directed DNA methylation (RdDM) pathway to buffer ploidy-driven transcriptional fluctuations. The jumping and insertion of transposable elements (TEs) can alter chromosome structure and gene expression, providing a driving force for species evolutionary diversity, while cytosine methylation modification can suppress the activity of transposons, thereby constraining their mobility [49]. Stable TE methylation (Figure 3B) supports an epigenetic silencing barrier hypothesis, wherein methylation preferentially immobilizes TEs to prevent transposition bursts, thereby safeguarding genome integrity—a mechanism potentially conserved across polyploid lineages. Integrating the results from Figure 2 and Figure 3, whole-genome duplication preferentially enhances CHH methylation near genes while maintaining overall epigenetic stability, suggesting that DNA methylation may help preserve genome stability and suppress transposable element activity after genome duplication.

**Figure 6:**
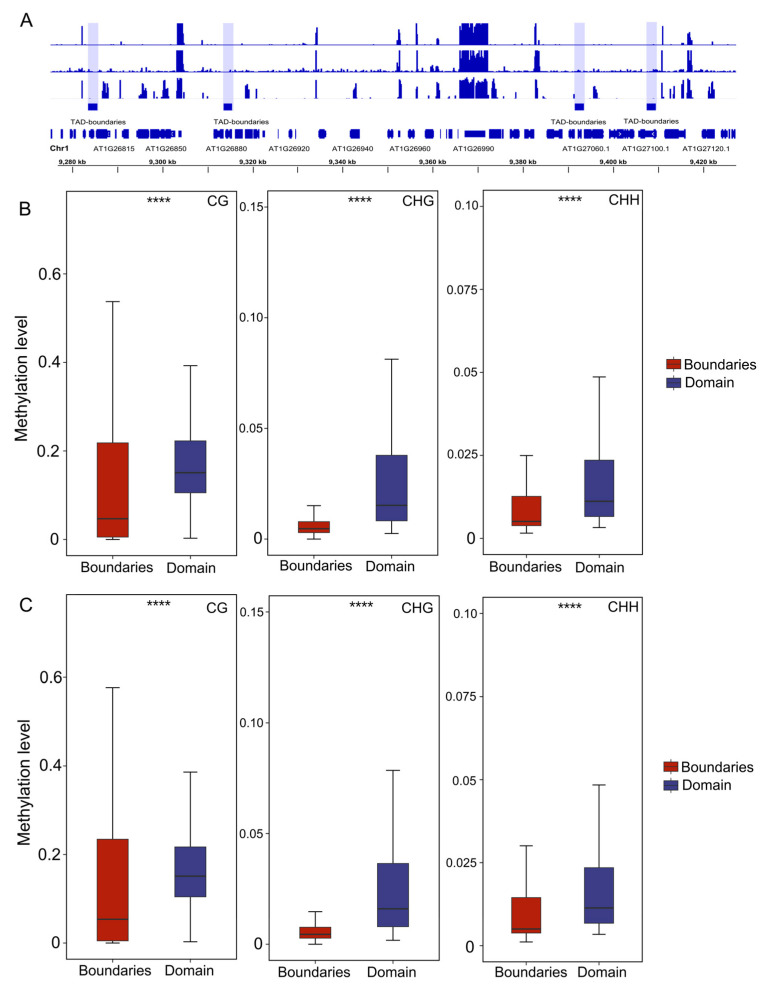
Interplay between DNA methylation and TADs during genome duplication. (**A**) IGV plot of methylation level (tracks from top to bottom are CG, CHG, CHH) and distribution of TADs. (**B**) DNA methylation level in TAD-like domain and boundaries of Ara 2X. (**C**) DNA methylation level in TAD-like domain and boundaries of Ara4X. **** *p* < 0.001 (*t* test).

The 3D genome plays critical roles in plant developmental regulation, stress adaptation, and genome stability [50,51]. In cotton, TAD architectures are implicated in subgenome dominance during evolution [52]. Soybean exhibits compartment reorganization and TAD remodeling following whole-genome polyploidization [53]. DNA methylation can regulate chromatin accessibility in plants and thereby influence the three-dimensional structure of the genome [54]. Our prior work in autotetraploid Arabidopsis revealed that 3D genome reorganization modulates gene expression through chromatin loop dynamics and histone modification patterns [9]. Our analysis reveals that whole-genome duplication (WGD)-induced changes in compartment interactions (Figure 5A) and conserved methylation at TAD boundaries (Figure 6B,C) suggest 3D genome reorganization may restrict methylation spread through physical insulation or chromatin loop anchoring, maintaining local epigenetic homeostasis. This provides novel insights into the “epigenetic-3D interplay” regulatory network in polyploid genomes. In summary, this study uncovers the dual role of DNA methylation during Arabidopsis polyploidization: maintaining stability while enabling local adaptive regulation.

## 4. Materials and Methods

### 4.1. Plant Materials and Growth Conditions

The autotetraploid Arabidopsis germplasm originated from our prior study [9], with growth conditions maintained as previously standardized. In short, seeds were surface sterilized with 75% ethanol for 10 min, rinsed three times with sterile water, and stratified at 4 °C for 48 h. They were then transferred to 1/2 MS solid medium. Cultivation conditions: growth chamber at 22 °C with a 16/8 h light/dark cycle and 65% humidity.

### 4.2. RNA-Seq Analysis

The optimized RNA-seq data analysis pipeline is structured as follows: Adapter sequences were first trimmed using fastp (v0.21.0) [55], followed by alignment of quality-controlled clean reads to the *Arabidopsis thaliana* reference genome using HISAT2 (v2.2.1) [56,57]. Functional annotation of genes employed clusterProfiler for GO enrichment analysis [58], where significantly enriched terms were identified using a corrected *p*-value threshold ≤ 0.05. Visualization of analytical results and gene expression clustering were accomplished using R packages including ggplot2 (v3.5.1), mfuzz (v2.62.0), and ClusterGVis (v0.1.2) [59].

### 4.3. WGBS and Analysis

We extracted genomic DNA from leaf tissues using a CTAB-based method with biological duplicates. DNA concentration was measured using an Agilent 2100 spectrophotometer. Subsequent bisulfite conversion (EZ DNA Methylation-Gold Kit, Zymo, Irvine, CA, USA) and library preparation (TruSeq DNA Methylation Kit, Illumina, San Diego, CA, USA) were followed by paired-end sequencing on an Illumina Novaseq 6000 platform, with all post-extraction procedures conducted by Oebiotech (Shanghai, China). For data analysis, fastp (v0.21.0) was used to filter raw data reads to obtain clean reads too, with parameters “-q 20 -u 30 -n 6 -c”. The high-quality reads were mapped to TAIR 10 and the B.rapa genome using Bismark (v0.23.1) software [60] with default parameters. The PCR duplicates were removed by deduplicate_bismark of Bismark software. The cytosine sites were identified on a whole-genome scale using bismark_methylation_extractor of Bismark software. To identify DMRs, DNA methylation levels were calculated for 300 bp sliding windows with 60 bp step sizes in the genome using methylKit v1.10.0 software [61]. The DMRs were selected based on the criteria of an adjusted *p* < 0.01 and methylation difference threshold percentage of 25, 15, and 10 for the CG, CHG, and CHH contexts, respectively. IGV browser [62] was employed to visualize the methylation level. The WGBS data generated in this study were deposited in the National Center for Biotechnology Information (NCBI) under accession number PRJNA1260687.

### 4.4. Hi-C Data Analysis

Raw Hi-C sequencing reads underwent adapter trimming and quality filtering with Trimmomatic v0.39 [63], followed by alignment to the TAIR10 genome using HiC-Pro v3.1.0 (MAPQ ≥ 30), with parameters “adapter.fa:3:30:10; SLIDINGWINDOW:4:20; MIN-LEN:50”. After removing duplicates and non-uniquely mapped reads, valid interactions were binned at different resolution and normalized via ICE software (v1.9.8). To identify 3D genome features, we performed principal component analysis (PCA) at 50 kb resolution to assign A/B compartments based on the first eigenvector’s sign, detected TAD-like domains using HiCExplorer [64]. The comparison of the interaction strength among compartments was accomplished by GENOVA [65]. Methylation levels at TAD boundaries/domains and shifted compartments were integrated via BEDTools [66]. TADCompare used to identify different TADs, consistent with prior work in Arabidopsis [67,68]. The identification of TADs and A/B compartments depends on the algorithms and resolution used, and different methods may yield slightly different results. We tried various software and parameter settings in our analysis and obtained consistent and reliable results.

## 5. Conclusions

In conclusion, this study provides the first comprehensive characterization of DNA methylation patterns in *Arabidopsis thaliana* following WGD. We revealed a marked upregulation of CHH methylation post-doubling, yet DNA methylation predominantly functions to maintain stable in changed 3D architectural during WGD. These findings advance our understanding of epigenetic mechanisms underlying polyploidization in plants, offering critical insights for future research on genome evolution and crop improvement. Future studies may investigate how methylation interacts with histone modifications and chromatin remodeling complexes to maintain transcriptional homeostasis in polyploid crops.

## Data Availability

All data used in this study have been uploaded to NCBI. Moreover, all other data are available from the corresponding author upon reasonable request.

## Supplementary Materials

The following supporting information can be downloaded at: https://www.mdpi.com/article/10.3390/plants14192959/s1, Figure S1: IGV plot of the DNA methylation levels of random gene and TE; Figure S2: GO enrichment of the CHH DMRs. Figure S3: Interaction matrix of diploid and autotetraploid *Arabidopsis thaliana*; Figure S4: The distribution of TAD is on chromosome 3; Figure S5: Comparison of TADs between hybrids and parents at 5 k resolution. The comparison results from TADCompare revealed five types: Split, Complex, Non-Differential, Merge, and Shifted; Table S1: Statistics of reads quantity and quality of DNA methylation sequencing data. Table S2: The quality of Hi-C reads in autotetraploid and wild-type Arabidopsis.

## Abbreviations

The following abbreviations are used in this manuscript:

Hi-C	High-resolution chromosome conformation capture
Ara	Arabidopsis thaliana
Bra	Brassica rapa
2x	diploid
4x	autotetraploid
TAD	topologically associating domain
DMGs	Different methylation genes
