# Supplementary material for: The Association Between DNA Methylation and Three-Dimensional Genome During Whole Genome Doubling in Arabidopsis thaliana
Source: Plants (Basel). 2025 Sep 24;14(19):2959. doi: 10.3390/plants14192959 (PMC12526026; doi:10.3390/plants14192959)
Supplement: Supplementary file 1 [file plants-14-02959-s001.zip › plants-3814480-supplementary.pdf]

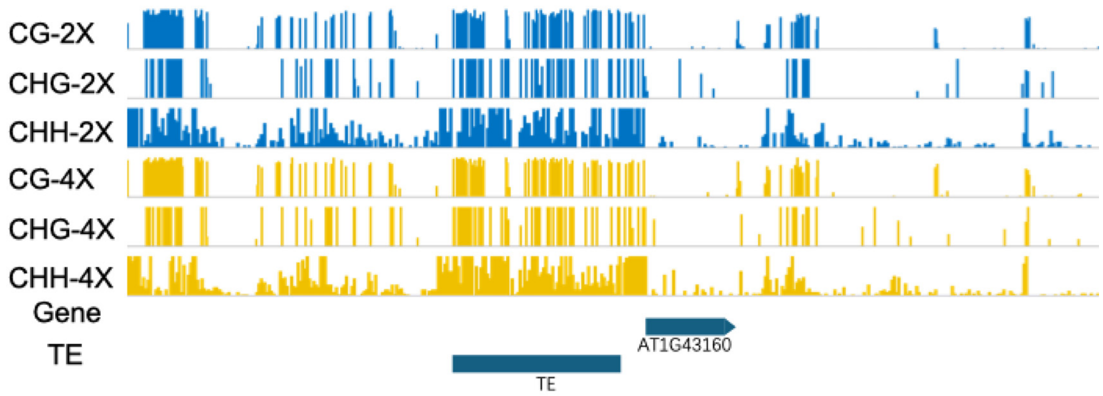

Figure S1 IGV plot of the DNA methylation levels of random gene and TE.

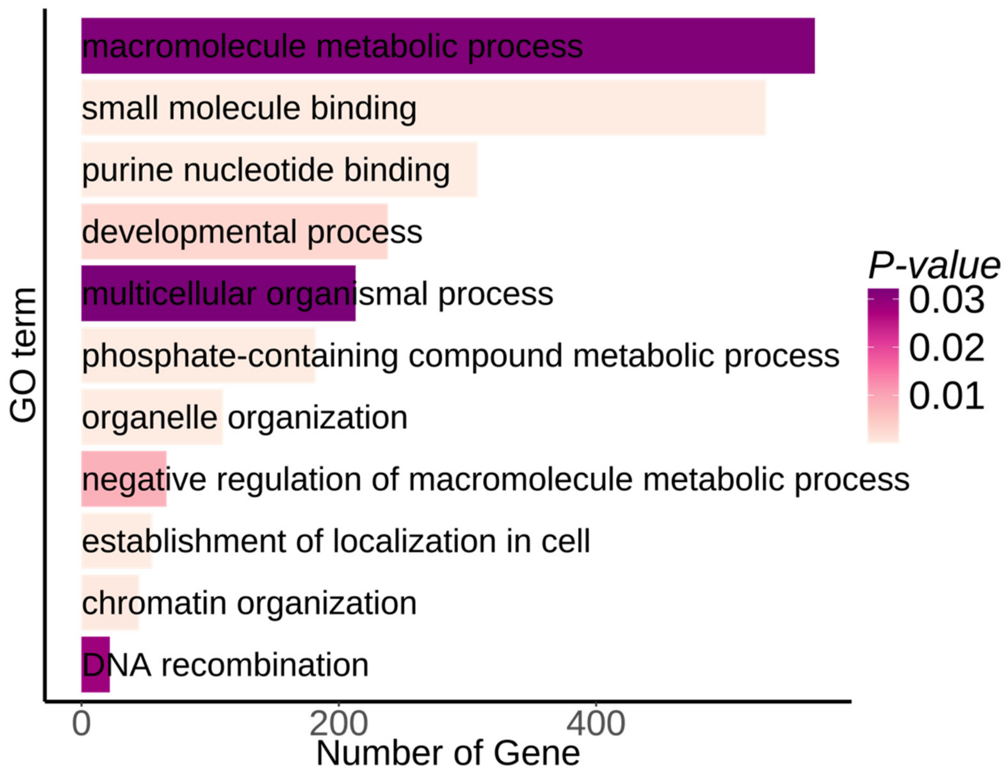

Figure S2 GO enrichment of the CHH DMRs.

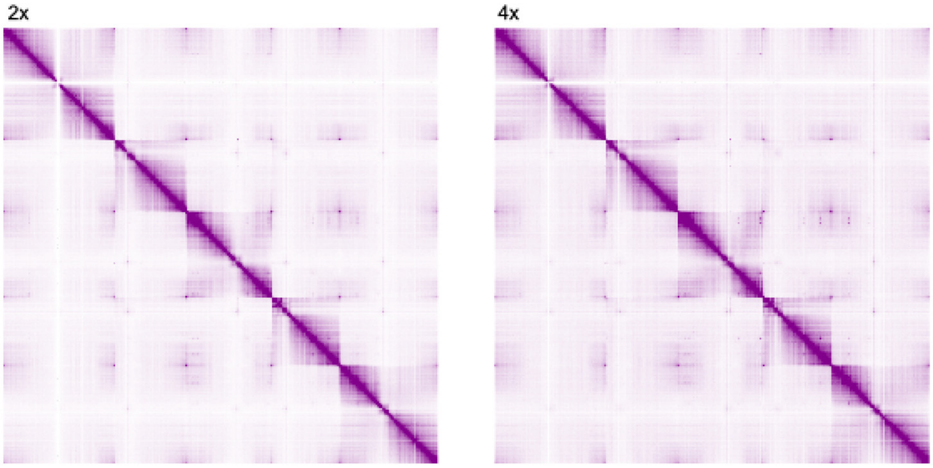

Figure S3 Interaction matrix of diploid and autotetraploid *Arabidopsis thaliana*

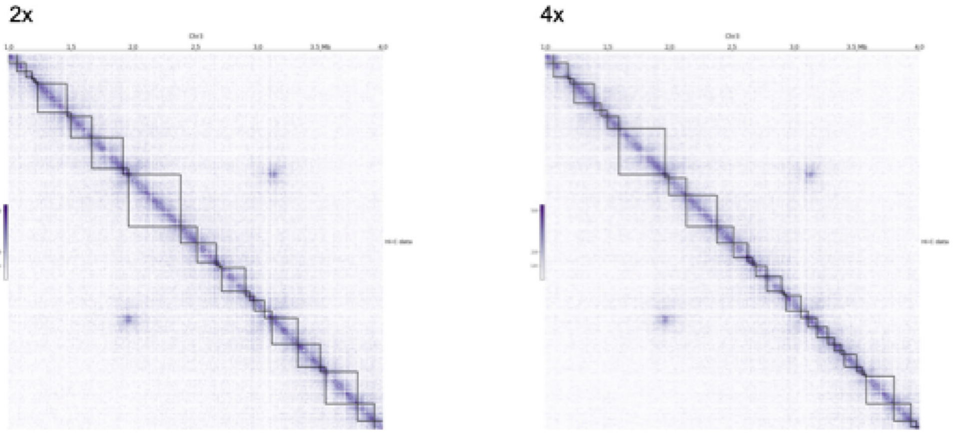

Figure S4 The distribution of TAD is on chromosome 3.

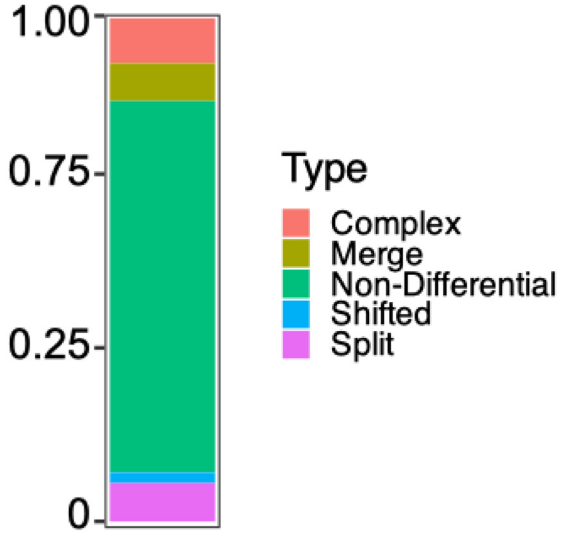

Figure S5. Comparison of TADs between hybrids and parents at 5 k resolution. The comparison results from TADCompare revealed five types: Split, Complex, Non-Differential, Merge, and Shifted

Table S1 Statistics of reads quantity and quality of DNA methylation sequencing data.

Samples	Ara2X	Ara4X
Clean Reads (M)	42,203,223	44,214,341
Q20 (%)	96.32	96.44
Q30 (%)	91.25	90.54
Average Depth (X)	35	33
Bisulfite Conversion Rate (%)	99.22	99.43
Mapping Rate (%)	70.21	72.3

Table S2 The quality of Hi-C reads in autotetraploid and wild type Arabidopsis.

Sample	Total raw reads	Total mapped reads	Valid 3C pairs	Total valid interaction
2x-Rep1	107,114,117	100,904,053	3,776,715	2,788,001
2x-Rep2	216,623,451	137,131,239	21,234,682	19,565,133
4x-Rep1	121,594,799	112,051,100	8,655,444	6,218,083
4x-Rep2	214,093,387	150,083,557	28,974,003	26,460,490
